# Increased risk of yellow fever infections among unvaccinated European travellers due to ongoing outbreak in Brazil, July 2017 to March 2018

**DOI:** 10.2807/1560-7917.ES.2018.23.11.18-00106

**Published:** 2018-03-15

**Authors:** Céline M Gossner, Joana M Haussig, Chiara de Bellegarde de Saint Lary, Kaja Kaasik Aaslav, Patricia Schlagenhauf, Bertrand Sudre

**Affiliations:** 1 European Centre for Disease Prevention and Control (ECDC), Stockholm, Sweden; 2 World Health Organization Collaborating Centre for Travellers’ Health, Epidemiology, Biostatistics, and Prevention Institute, University of Zürich, Switzerland

**Keywords:** Brazil, vector-borne infections, viral infections, yellow fever, yellow fever virus, outbreaks

## Abstract

Since December 2016, Brazil has faced a large outbreak of yellow fever with ca 1,500 confirmed human cases. In the first 2 months of 2018, Brazil reported almost as many cases as in 2017 as a whole. In these 2 months, five imported cases were reported among unvaccinated European travellers. Three had travelled to Ilha Grande, a popular destination among European tourists. Physicians and European travellers visiting Brazil should follow yellow fever vaccination recommendations.

Since December 2016, Brazil has faced a large outbreak of yellow fever (YF) [1]. The recent increase in magnitude of this outbreak, both in terms of geographical spread and in number of cases, poses an increased risk of YF infection to unvaccinated European travellers to Brazil as evidenced by recent reports of five imported cases to Europe in the past 2 months.

## Ongoing outbreak in Brazil

Yellow fever is an arboviral disease of primates transmitted by mosquitoes of the *Aedes* and *Haemagogus* species. In Brazil, YF is endemic in large parts of the country. Historically, the regions from where most cases were reported were forested and rural areas such the hydrographic basins of the Amazon, Araguaia, Tocantins and Paraná rivers [2].

**Figure 1:**
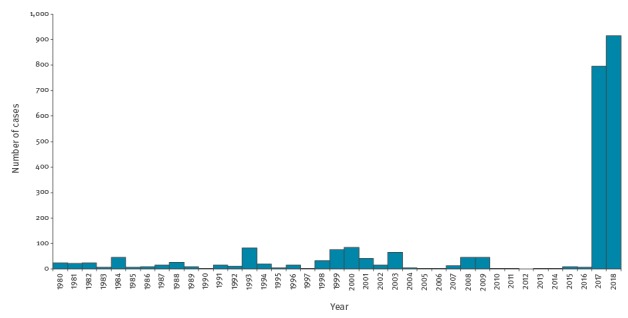
Distribution of confirmed human cases of yellow fever by year, Brazil, 1980–13 March 2018 (n = 2,509)

In 2014/15, YF virus emerged at the periphery of endemic areas with the detection of non-human primate (NHP) cases [1]. In Brazil, yellow fever surveillance is set up from July to June the following year. During the surveillance period 2016/17 (July 2016 to June 2017), the virus spread geographically, reaching nine states in central and south-eastern Brazil (i.e. Distrito Federal, Espírito Santo, Goiás, Mato Grosso, Minas Gerais, Pará, Rio de Janeiro, São Paulo and Tocantins) and causing 779 human cases including 262 deaths (Figure 1) [1].

Data as at 13 March 2018.

**Figure 2:**
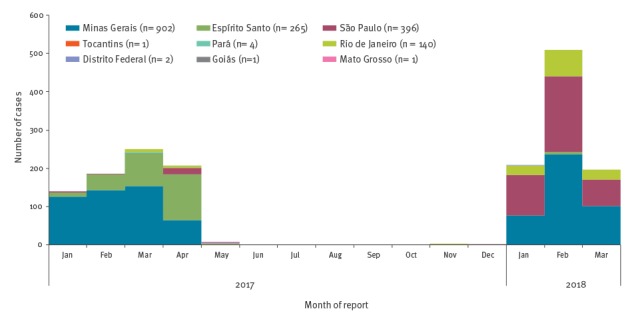
Distribution of confirmed human cases of yellow fever by month of reporting and by state, Brazil, January 2017–13 March 2018 (n = 1,712)

In addition, 1,659 epizootics (clusters of cases detected among NHPs) in NHPs were reported, causing the death of more than 2,000 animals. After a decline in incidence of human cases from April to May 2017, and very few cases reported between June and November 2017, a second wave of the outbreak started in December 2017 (Figure 2) [3].

Data as at 13 March 2018.

**Figure 3:**
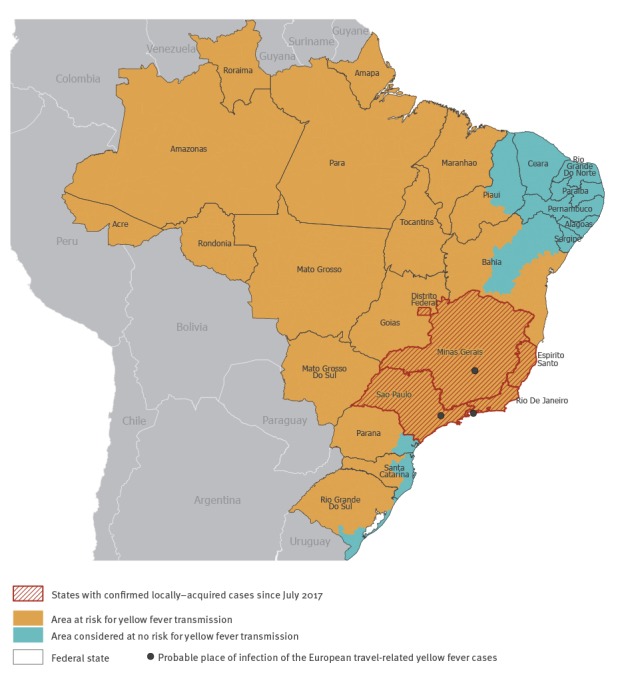
Distribution of confirmed yellow fever cases by state and areas at risk, Brazil, July 2017–13 March 2018

From July 2017 to 13 March 2018, 920 confirmed human cases including 300 deaths were reported; most cases occurred in January and February 2018 [3]. In comparison, during the same period in 2016/17, there were ca 610 confirmed human cases. Between July 2017 and 13 March 2018, five states reported YF cases: Minas Gerais (n = 415), São Paulo (n = 376), Rio de Janeiro (n = 123), Espírito Santo (n = 5) and Distrito Federal (n = 1). In the same period, there were 617 epizootics, the majority of them in the State of São Paulo (n = 502) (Figure 3). While the increase in the number of human cases started in December 2017, the upsurge of epizootics has been ongoing since mid-September 2017. This situation is consistent with the fact that NHPs are more exposed to mosquitoes compared with humans, hence they are commonly used as sentinels for early detection of YF transmission [2,4].

Source [18].

The detection of epizootic events in the States of São Paulo and Rio de Janeiro with close vicinity to two highly populated metropolitan regions remains of concern [5,6]. It highlights an increased likelihood of peri-urban or urban cycles of YF transmission, which in megacities such as São Paulo and Rio de Janeiro could lead to very large outbreaks and consequently higher risk of exposure of international travellers.

On 15 February 2018, YF virus was identified in *Ae. albopictus* mosquitoes captured in two municipalities (Ituêta and Alvarenga) in Minas Gerais state [7]. In a laboratory setting, studied Brazilian populations of *Ae. albopictus* have proven to be a competent vector for YF virus which underlines the risk of potential spread of the virus through this mosquito species and hypothetically the risk of sustained urban cycle through *Ae. albopictus* [8].

Brazil is currently conducting mass vaccination campaigns to increase the vaccination coverage in Bahia, Rio de Janeiro and São Paulo states. As at 13 March 2018, 17.5 million people have been vaccinated, with vaccination coverages of 54%, 69% and 95% in the three states respectively [3].

## Travellers and imported cases

According to the International Air Transport Association, between 2012 and 2016, there were on average 2.4 million travellers arriving every year from Brazil to Europe [9]. The number of travellers departing from Brazil to Europe is highest between May and September. In addition, a smaller peak is observed from December to January. Between December 2017 and May 2018, the highest risk period of YF infection in Brazil (based on the seasonality of the vector activity and the YF epidemic in the previous seasons), we estimated that there would be around 1.2 million travellers returning from Brazil to Europe.

The Carnival, one of the largest international mass gatherings in Brazil, took place between 9 and 14 February 2018. This event brings together several millions of people from throughout the country and from abroad. In its rapid risk assessment published on 18 January 2018, the European Centre for Disease Prevention and Control (ECDC) highlighted the possibility of an increase in number of travel-related cases among unvaccinated Europeans [10]. Since January 2018, five unvaccinated European travellers were infected in Brazil (Table).

**Table:** Overview of yellow fever cases among European and non-European travellers returning from Brazil, 2018

Reporting country	Likely place of exposure	Period of exposure	Sex	Age	Vaccination status	Outcome	Source and date of publication
**European travellers**							
The Netherlands	Mairiporã, SP	Dec 2017–Jan 2018	Male	46 years	Unvaccinated	Recovered	15 Jan 2018 [17]
France	MG	NA	Female	NA	Unvaccinated	Recovered	14 Feb 2018 [17]
Romania	Rio de Janeiro city and Ilha Grande, RJ	Feb 2018	Male	NA	Unvaccinated	NA	28 Feb 2018 [18]
Switzerland	Ilha Grande, RJ	Feb 2018	Male	44 years	Unvaccinated	Fatal	28 Feb 2018 [18]
United Kingdom	Rio de Janeiro city and Ilha Grande, RJ	Feb–Mar 2018	NA	NA	NA	NA	Feb 2018 [19]
**Non-European travellers**							
Argentina	Ilha Grande, RJ and Ilhabela, SP	Jan–Feb 2018	Female	45 years	Unvaccinated	Recovered	Mar 2018 [12]
Argentina	Ilha Grande, RJ	Feb 2018	Male	28 years	Unvaccinated	Recovered	Mar 2018 [12]
Argentina	Salvador de Bahía, BA and Ouro Preto, MG and Río de Janeiro, RJ	Dec 2017–Feb 2018	Female	22 years	Unvaccinated	Recovered	Mar 2018 [12]
Chile	Ilha Grande, RJ	NA	Male	35 years	Unvaccinated	Fatal	20 Feb 2018 [20]
Chile	Ilha Grande, RJ	NA	Male	20 years	Unvaccinated	Fatal	20 Feb 2018 [20]
Chile	Ilha Grande, RJ	NA	Male	NA	NA	Alive	20 Feb 2018 [20]

BA: Bahia; MG: Minas Gerais; RJ: Rio de Janeiro; SP: São Paulo; NA: not available.

### Imported cases to Europe

Between 1999 and 2017, only seven travel-associated YF cases have been reported in the scientific literature among unvaccinated European travellers, none of them returning from Brazil [10].

The 2018 cases were reported by France, the Netherlands, Romania, Switzerland and the United Kingdom. Age was available for two of them who were in their mid-40s. Three cases were male, one was female and information on sex was unknown for the remaining case. None of the five cases was vaccinated, one died. One case travelled to Mairiporã, São Paulo state, another to Minas Gerais and the other three cases reported a travel to Ilha Grande in the Angra dos Reis municipality, an island situated off the coast of Rio de Janeiro. The island is a popular tourist destination and prior to 2016, Ilha Grande was not considered as a risk area for YF. Between 1 January and 13 March 2018, Brazil reported 40 YF cases, including 14 deaths, from Angra dos Reis municipality, accounting for the highest case numbers in Rio de Janeiro state [11].

### Imported cases within South America

Six travel-related cases among non-European travellers were reported in February 2018, three by Argentina and three by Chile. For five cases age was available, they were between 20 and 45 years old, the majority were men (n = 4), all five for whom information was available were unvaccinated, two cases died. Five of the six cases reported a travel to Ilha Grande, Angra dos Reis municipality [12,13].

## Discussion and conclusions

The increase in YF transmission in central and south-eastern Brazil has resulted in the occurrence of travel-related cases among unvaccinated European and South American travellers who visited popular tourist destinations where the virus is circulating intensely. The recent change in the YF epidemiological situation in Brazil seems to be particularly noticeable in Ilha Grande a common travel destination and the place of exposure of the majority of the travel-related cases.

The YF cases in non-vaccinated tourists highlight the relevance of YF vaccination of travellers to risk areas for YF and the need for a comprehensive pre-travel advice to assess the vaccination status. These cases also emphasize that the epidemiological situation in each region is or may be evolving rapidly and that pre-travel advice should be completed in a timeframe allowing to consider the latest epidemiological situation while providing enough time for an efficient vaccination protection.

## Risk of spread of yellow fever virus in Europe

The probability of local YF transmission in continental Europe following introduction of the virus by a viraemic traveller is currently considered very low; *Ae. aegypti*, the main vector of YF virus in urban settings, is absent in continental Europe. It is established in the outermost region of Madeira and was recently introduced in the Canary Islands [14]. During the European winter season the weather conditions are, however, not favourable to vector activity. *Ae. albopictus*, which is largely established in the southern part of continental Europe, was shown to be a competent vector for YF virus in laboratory settings, though YF transmission via *Ae. albopictus* has never been observed in nature [14,15].

## Public health implications for Europe

To inform European public health authorities about the YF risk for travellers, ECDC has published several risk assessments [10]. Through the VectorNet project, ECDC is monitoring the presence/absence of *Ae. aegypti* and *Ae. albopictus* in Europe [14].

The two consecutive waves of transmission, both of a previously undocumented magnitude, have caused the World Health Organization and the Brazilian health authorities to review the areas considered at risk in Brazil. European health practitioners should remain attentive to the evolution of the epidemic and related updates in areas at risk and ensure adequate vaccination of their patients.

The last urban YF outbreak in Brazil took place in 1942 in Acre [16]; since then the risk of an urban cycle becoming established in cities such as São Paulo and Rio de Janeiro has never been as high as at present. The live-attenuated virus YF vaccine is safe, effective, and inexpensive and provides life-long immunity. The vaccine thus remains the most important means of preventing infections and controlling YF outbreaks [17]. ECDC encourages European countries to strengthen their awareness campaigns for travellers to Brazil to ensure that they are vaccinated if travelling to at-risk areas, in accordance with respective national recommendations (Figure 3) [18]. Travellers with contraindication for vaccination should seek medical advice before departure. In addition, it is important that clinicians are alerted to the ongoing outbreak and are able to early diagnose YF in travellers with compatible clinical symptoms. Finally, to protect themselves from YF infection and other arboviral diseases occurring in Brazil (e.g. dengue), travellers should avoid mosquito bites using chemical and/or mechanical barriers such as mosquito repellent, long-sleeved tee shirts and long trousers and bed nets.

## References

[1] Brazil Ministry of Health. Febre Amarela: Situação Epidemiológica / Dados. [Yellow fever. Epidemiological situation / Data]. Brasília: Ministry of Health. [Accessed 6 Mar 2018]. Portuguese. Available from: http://portalms.saude.gov.br/saude-de-a-z/febre-amarela-sintomas-transmissao-e-prevencao/situacao-epidemiologica-dados

[2] Cavalcante KR, Tauil PL. Epidemiological characteristics of yellow fever in Brazil, 2000-2012. Epidemiol Serv Saude. 2016;25(1):11-20. 27861674 10.5123/S1679-49742016000100002

[3] Brazil Ministry of Health. Informe n° 17 | 2017/2018 - Monitoramento do Período Sazonal da Febre Amarela Brasil – 2017/2018. [Report 17. 2017/2018. Monitoring the season of yellow fever in Brazil – 2017/2018]. Brasília: Ministry of Health. 13 Mar 2018. Portuguese. Available from: http://portalarquivos2.saude.gov.br/images/pdf/2018/marco/14/Informe-FA-17.pdf

[4] Saad LD, Barata RB. Yellow fever outbreaks in São Paulo State, Brazil, 2000-2010. Epidemiol Serv Saude. 2016;25(3):531-40. 27869924 10.5123/S1679-49742016000300009

[5] Prefeitura de São Paulo. Parques das Zonas Sul e Oeste da capital são fechados por medida de precaução e vacinação será intensificada. [Parks in the southern and western regions are closed as precautionary measure and vaccination will be intensified]. São Paulo: Prefeitura de São Paulo. [Accessed 6 Mar 2018]. Portuguese. Available from: http://www.capital.sp.gov.br/noticia/parques-das-zonas-sul-e-oeste-da-capital-sao-fechados-por-medida-de-precaucao-e-vacinacao-sera-intensificada

[6] Globo.com. [Internet]. SP tem 15 parques fechados por prevenção contra a febre amarela; veja lista. [15 parks closed in São Paulo as prevention measure against yellow fever; see list]. São Paulo: Globo. [Accessed 6 Mar 2018]. Portuguese. Available from: https://g1.globo.com/sao-paulo/noticia/sp-tem-15-parques-fechados-por-prevencao-contra-a-febre-amarela-veja-lista.ghtml

[7] World Health Organization (WHO). Yellow fever – Brazil, Disease outbreak news. Geneva: WHO; 27 Feb 2018. Available from: http://www.who.int/csr/don/27-february-2018-yellow-fever-brazil/en/

[8] Couto-Lima D Madec Y Bersot MI Campos SS Motta MA Santos FBD Potential risk of re-emergence of urban transmission of Yellow Fever virus in Brazil facilitated by competent Aedes populations. Sci Rep. 2017;7(1):4848. 10.1038/s41598-017-05186-3 28687779 PMC5501812

[9] International Air Transport Association (IATA). Market Intelligence Services. Geneva: IATA. [Accessed 7 Mar 2018]. Available from: http://www.iata.org/services/statistics/intelligence/Pages/market-intelligence.aspx

[10] European Centre for Disease Prevention and Control (ECDC). Outbreak of yellow fever in Brazil, Second update, 18 January 2018. Stockholm: ECDC; 2018. Available from: https://ecdc.europa.eu/sites/portal/files/documents/18-01-2018-RRA-UPDATE-2-Yellow-fever-Brazil.pdf

[11] Rio de Janeiro Government. Informe Epidemiológico – Febre Amarela, 13 Mar 2018. [Epidemiological report – Yellow fever, 13 Mar 2018]. Rio de Janeiro: Rio de Janeiro Government. 13 Mar 2018. Portuguese. Available from: http://www.febreamarelarj.com.br/comum/code/MostrarArquivo.php?C=NDY%2C

[12] Argentina Ministry of Health. Boletín Integrado de Vigilancia. N° 401 – SE 09 – Marzo de 2018. Monitoring bulletin. N 401 – SE 09. March 2018]. Buenos Aires: Ministry of Health; 5 Mar 2018. Spanish. Available from: http://www.msal.gob.ar/index.php/home/boletin-integrado-de-vigilancia

[13] ProMED-mail. PRO/ESP> Fiebre amarilla - Argentina: (BA) ex Brasil, turista no vacunada. 20180307.5671622. [PRO/ESP> Yellow fever - Argentina: (BA) ex Brasil, unvaccinated tourist. 20180307.5671622]. 7 Mar 2018. Spanish. Available from: http://www.promedmail.org/post/5671622

[14] European Centre for Disease Prevention and Control (ECDC) and European Food Safety Authority (EFSA). VectorNet: A European network for sharing data on the geographic distribution of arthropod vectors, transmitting human and animal disease agents. Aedes albopictus current known distribution. Stockholm: ECDC; Jan 2018. Available from: https://ecdc.europa.eu/en/publications-data/aedes-albopictus-current-known-distribution-january-2018

[15] Amraoui F Vazeille M Failloux AB French Aedes albopictus are able to transmit yellow fever virus. Euro Surveill. 2016;21(39):30361. 10.2807/1560-7917.ES.2016.21.39.30361 27719755 PMC5069433

[16] Bacha HA, Johanson GH. Yellow fever. Rev Assoc Med Bras (1992). 2017;63(4):291-2. PMID: 28614526. http://dx.doi.org/10.1590/1806-9282.63.04.291 28614526

[17] World Health Organization (WHO). Yellow fever mass vaccination campaigns using fractional dose, Extract from report of GACVS meeting of 30 Nov -1 Dec 2016, published in the WHO Weekly Epidemiological Record on 13 Jan 2017. Geneva: WHO. [Accessed 6 Mar 2018]. Available from: http://www.who.int/vaccine_safety/committee/topics/yellow_fever/Dec_2016/en/

[18] World Health Organization (WHO). Yellow fever vaccination recommendations in the Americas, Geneva: WHO. [Accessed 7 Mar 2018]. Available from: http://www.who.int/ith/YF-Vaccination-map-americas.jpg?ua=1

[19] Public Health England (PHE). Yellow fever reported in traveller returning from Brazil. London: PHE; 15 Mar 2018. Available from: https://www.gov.uk/government/news/yellow-fever-reported-in-traveller-returning-from-brazil

[20] ProMED-mail. PRO/AH/EDR> Yellow fever - Americas (15): Brazil (RJ). 20180222.5643503. 20 Feb 2018. Available from: http://www.promedmail.org/post/5643503.

